# Stroma AReactive Invasion Front Areas (SARIFA) predict poor survival in adenocarcinomas of the stomach and gastrooesophageal junction: a validation study

**DOI:** 10.1007/s00428-024-03826-4

**Published:** 2024-05-15

**Authors:** Dita Ulase, Hans-Michael Behrens, Christoph Röcken

**Affiliations:** Department of Pathology, University Hospital Schleswig-Holstein, Campus Kiel, Arnold-Heller-Str. 3, Building U33, 24105 Kiel, Germany

**Keywords:** Gastric cancer, Histopathology, Invasive margin, Prognosis

## Abstract

Recently, the presence of “Stroma AReactive Invasion Front Areas” (SARIFA) has been described as a promising adverse prognostic factor in gastric cancer. However, the validity of this approach still needs to be tested. The aim of this study was to independently assess the utility of the proposed method in a well-characterised cohort of primary resected adenocarcinomas of stomach and gastrooesophageal junction (*n* = 392). SARIFA status was analysed on routine slides of resection specimens. Cases were divided into SARIFA-positive and negative groups and analysed in relation to clinicopathological and survival data. SARIFA positivity was found in 15.1% (*n* = 59) cases and was significantly associated with Lauren phenotype (*p* < 0.001), pT (*p* = 0.001), pN (*p* = 0.018), UICC stage (*p* = 0.031), tumour budding (*p* = 0.002), overall survival (*p* < 0.001) and cancer-specific survival (*p* < 0.001). SARIFA-positive tumours had a worse prognosis in the multivariate setting (HR = 1.847, 95% CI: 1.300–2.624, *p* = 0.001). SARIFA status is an independent prognostic factor in gastric cancer, in particular in locally advanced tumours.

## Introduction

Adenocarcinoma of the stomach and gastrooesophageal junction (GC) remains a global health concern. According to GLOBOCAN data, GC ranks as the fourth leading cause of cancer-related deaths worldwide, with an estimated 769,000 deaths in 2020 [1]. Despite the progress in diagnostic approaches and available treatment regimens, the prognosis of GC is poor. The traditional TNM staging system, i.e. depth of invasion, regional nodal involvement and presence of distant metastases, is the gold standard in the prediction of prognosis in GC. However, GC is a highly heterogeneous malignancy in terms of its phenotype, genotype and clinical outcome.

As molecular subtyping is time- and cost-consuming and thus not yet applicable for routine diagnostics, additional prognostic indicators are needed to improve risk stratification for patients with GC. Several morphological patterns have been suggested as prognostic factors in GC, including tumour budding, poorly differentiated clusters, stroma maturity or tumour-stroma ratio [2–4]. None of them have been introduced into clinical practice.

Recently, the presence of “Stroma AReactive Invasion Front Areas” (SARIFA) has been described as a promising morphology-based prognostic factor in colorectal cancer (CRC) and GC [5, 6]. SARIFA is defined as an area at the invasive margin in which a tumour gland or a group of at least 5 tumour cells directly approach adipocytes without a stromal reaction or a histiocytic reaction [5]. The same phenomenon, described as a tumour-adipose feature (TAF), has been identified in another study of CRC by using deep learning systems [7]. Authors have demonstrated that the presence of SARIFA/TAF is associated with adverse prognosis. Adipose tissue is one of the components of the tumour microenvironment (TME) that supports cancer growth and spread. Tumour cells that are surrounded by adipocytes are able to switch their metabolism from glycolysis to lipid-dependent energy production [8]. SARIFA appears to be related to alterations in lipid metabolism, in particular, the upregulation of fatty-acid binding protein 4 (FABP4) and CD36 [6]. These molecules maintain the uptake of fatty acids in cells, which are used for energy metabolism, synthesis of membranes and lipid-derived cell signalling molecules, thus promoting tumour growth [8]. Furthermore, lipid-rich TME shows an immunosuppressive nature. CD36-mediated uptake of fatty acids, lipid peroxidation and ferroptosis reduces functions of intratumoural CD8+ effector T cells and impairs antitumour immunity [9].

To our knowledge, the role of SARIFA in GC has been investigated only by one author collective, and thus, the evidence base for SARIFA as a biomarker is limited. Therefore, the aim of this study was to independently evaluate SARIFA status and its reliability and potential prognostic value in an independent, large, well-characterised Western patient cohort of GCs.

## Materials and methods

### Study population

This retrospective study considered all patients with adenocarcinoma of the stomach and gastrooesophageal junction who have undergone primary total gastrectomy or partial gastric resection from 1997 to 2009 at the University Hospital Schleswig-Holstein, Kiel, Germany. The exclusion criteria were as follows: (1) preoperative chemoradiation or perioperative chemotherapy received and (2) intramucosal (pT1a) tumours. Haematoxylin and eosin (H&E) stained whole tissue sections of the resection specimens were reviewed, and one representative slide per case was used for further analysis. Clinicopathological characteristics were collected from previous records, including sex, age at diagnosis, tumour localization, type by Laurén, pathologic stage (pTNM) according to the 8th edition of the UICC guidelines [10], status of resection lines (pR), lymph node ratio and lymphatic or venous invasion (LVI). For survival analysis, the date of surgery and date of death or last follow-up was used. Data about the causes of death were previously collected from the Cancer Registry of Schleswig-Holstein. Microsatellite instability (MSI), Epstein-Barr virus (EBV), HER2 and MET status were available from past studies of the cohort [11–14]. Tumour budding score was assessed according to the ITBCC criteria [15, 16] and categorised into Bd0 to Bd3 as previously described [17].

### Assessment of SARIFA

The assessment of SARIFA was performed according to the criteria given by the original authors. Briefly, SARIFA was defined as an area at the invasive margin where a tumour gland or a group of at least 5 tumour cells directly approached adipocytes without a stromal reaction (fibroblastic proliferation, collagen formation) or a histiocytic reaction [5]. When the invasive margin did not extend to adventitia (e.g. pT2-stage), direct contact of tumour cell clusters to local tissue (submucosa or muscularis propria) was assessed, as proposed by Grosser et al. [6]. In this study, the invasive margin was defined as the outermost cell layers at the invading edge of the tumour in a given tissue section. Tumours were classified as SARIFA-positive if at least a single SARIFA was found. Challenging cases were resolved by consensus review.

### Statistical analysis

Data were analysed using SPSS 25.0.0.2 (IBM Corporation, New York, USA). A significance level of *p* < 0.05 was chosen. Associations with demographic and clinicopathological variables were analysed using cross-tabulation analysis and Kendall’s tau test for ordinal variables or Fischer’s exact test for non-ordinal variables. Spearman’s rank correlation coefficient was calculated to determine the correlation between SARIFA and tumour budding. Overall survival (OS) and cancer-specific survival (CSS) were defined as the time from the date of surgery until death due to any cause and death due to GC, respectively. Survival curves were estimated using the Kaplan–Meier method and compared using the log-rank test. Multivariate survival analysis was performed using a backward stepwise Cox regression model and included all covariates that were identified at a level of *p* < 0.100 in the univariate analysis. To account for the false discovery rate, the Benjamini–Hochberg (Simes) method was applied to the pool of all *p*-values of this study (*n* = 24) [18]. All the *p*-values are given uncorrected. Those *p*-values which have lost significance are marked accordingly.

## Results

### Associations with clinicopathological characteristics

**Fig. 1:**
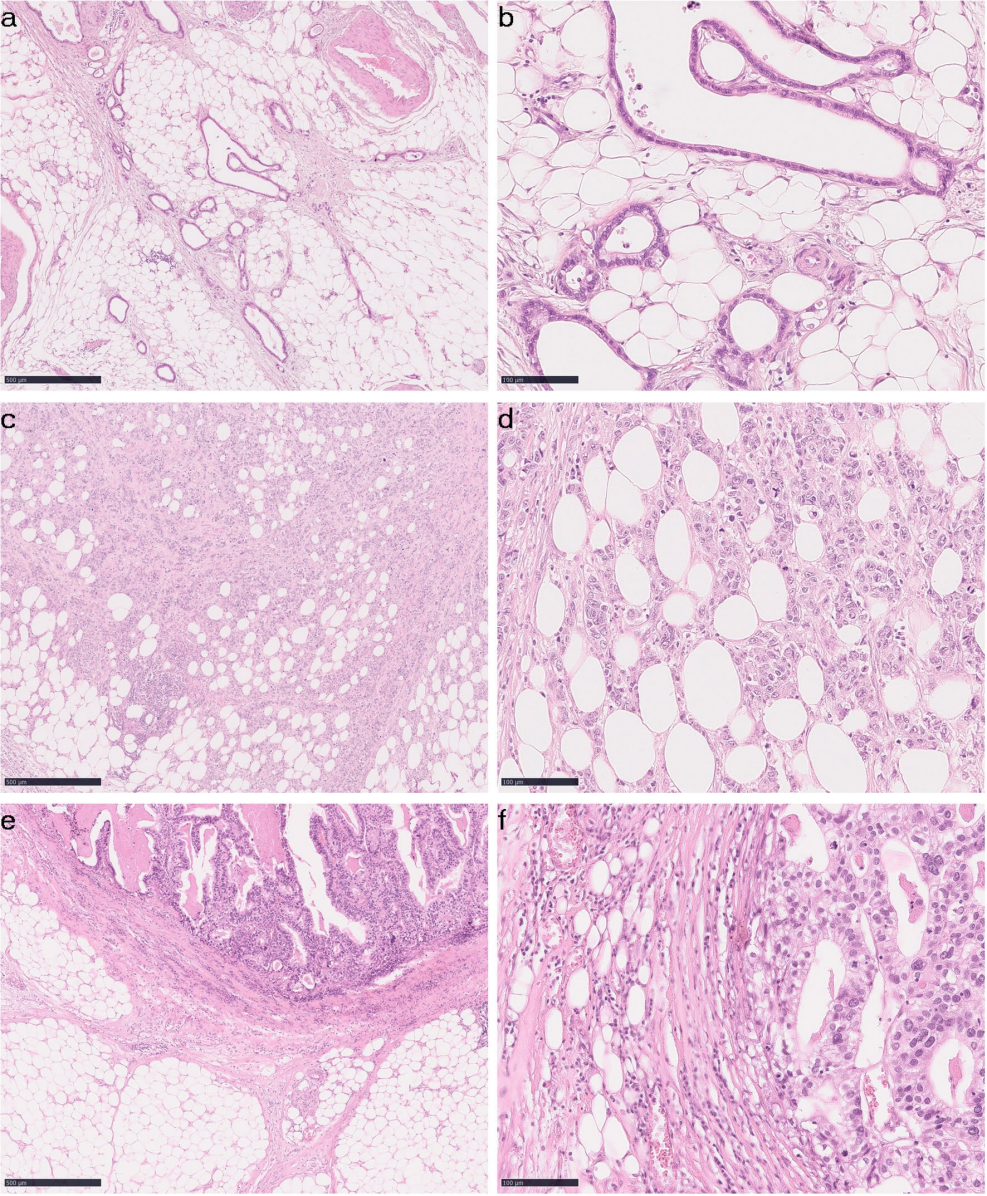
SARIFA in gastric cancer and cancer of gastroesophageal junction. Representative tissue sections of SARIFA-positive (**a**–**d**) and SARIFA-negative (**e**–**f**) tumours. Haematoxylin and eosin stain, original magnification 50× (scale bar represents 500 μm) and 200× (scale bar represents 100 μm)

In this cohort of 392 GCs, 15.1% cases (*n* = 59) were SARIFA-positive. Examples of SARIFA-positive and SARIFA-negative GC are found in Fig. 1. Associations between the status of SARIFA and clinicopathological variables are summarised in Table 1. SARIFA positivity was more commonly observed in cases with deeper invasion, positive nodal status and, thus, advanced tumour stage. There was a very weak, positive correlation between SARIFA status and tumour budding (*r*_*s*_ = 0.160, *n* = 359, *p* = 0.002). No statistically significant associations could be detected between SARIFA status and patients’ sex, age, tumour localization, tumour grade, presence of distant metastases, lymph node ratio, lymphatic or vascular invasion, status of resection lines, EBV, MSI, HER2 and MET status. Interestingly, among 31 GCs with *MET* amplification, 8 cases (25.8%) were SARIFA-positive, although the association was not statistically significant.

**Table 1:** Clinicopathological patient characteristics and correlation with SARIFA status

Characteristics	SARIFA						
	Valid/missing		negative		positive		*p*-value
	*n*	(%)	*n*	(%)	*n*	(%)	
Sex	392/0						0.107^a^
Male	246	(62.8)	203	(82.5)	43	(17.5)	
Female	146	(37.2)	130	(89.0)	16	(11.0)	
Age	392/0						0.089^a^
<68 years	189	(48.2)	167	(88.4)	22	(11.6)	
≥68 years	203	(51.8)	166	(81.8)	37	(18.2)	
Localization	390/2						0.095^a^
Proximal stomach	128	(32.8)	103	(80.5)	25	(19.5)	
Distal stomach	262	(67.2)	229	(87.4)	33	(12.6)	
Laurén phenotype	392/0						0.001^a^
Intestinal	219	(55.9)	185	(84.5)	34	(15.5)	
Diffuse	103	(26.3)	93	(90.3)	10	(9.7)	
Mixed	25	(6.4)	14	(56.0)	11	(44.0)	
Unclassified	45	(11.5)	41	(91.1)	4	(8.9)	
Grade (intestinal type only)	219/0						1.000^a^
Low (G1/G2)	90	(41.1)	76	(84.4)	14	(15.6)	
High (G3)	129	(58.9)	109	(84.5)	20	(15.5)	
pT category	392/0						0.001^a^
pT1b/pT2	89	(22.7)	85	(95.5)	4	(4.5)	
pT3/pT4	303	(77.3)	248	(81.8)	55	(18.2)	
pN category	391/1						0.018^a*^
pN0	112	(28.6)	103	(92.0)	9	(8.0)	
pN+	279	(71.4)	230	(82.4)	49	(17.6)	
pM category	392/0						0.856^a^
M0	321	(81.9)	273	(85.0)	48	(15.0)	
M1	71	(18.1)	60	(84.5)	11	(15.5)	
UICC stage	391/1						0.031^b*^
IA/IB	62	(15.8)	60	(96.8)	2	(3.2)	
IIA/IIB	86	(22.0)	74	(86.0)	12	(14.0)	
IIIA/IIIB/IIIC	172	(44.0)	139	(80.8)	33	(19.2)	
IV	71	(18.2)	60	(84.5)	11	(15.5)	
Lymph node ratio	391/1						0.090^a^
Low (<0.189)	189	(48.3)	167	(88.4)	22	(11.6)	
High (≥0.189)	202	(51.7)	166	(82.2)	36	(17.8)	
pR status	388/4						0.821^a^
pR0	344	(88.7)	294	(85.5)	50	(14.5)	
pR1/pR2	44	(11.3)	37	(84.1)	7	(15.9)	
L category	374/18						0.186^a^
L0	178	(47.6)	157	(88.2)	21	(11.8)	
L1	196	(52.4)	163	(83.2)	33	(16.8)	
V category	372/20						0.078^a^
V0	336	(90.3)	291	(86.6)	45	(13.4)	
V1	36	(9.7)	27	(75.0)	9	(25.0)	
Tumour budding	359/33						0.002^b^
Bd0	84	(23.4)	78	(92.9)	6	(7.1)	
Bd1	63	(17.5)	55	(87.3)	8	(12.7)	
Bd2	24	(6.7)	20	(83.3)	4	(16.7)	
Bd3	188	(52.4)	148	(78.7)	40	(21.3)	
HER2 status	368/24						0.136^a^
Negative	333	(90.5)	279	(83.8)	54	(16.2)	
Positive	35	(9.5)	33	(94.3)	2	(5.7)	
MET status	384/8						0.115^a^
Negative	353	(91.9)	302	(85.6)	51	(14.4)	
Positive	31	(8.1)	23	(74.2)	8	(25.8)	
EBV status	392/0						1.000^a^
Negative	373	(95.2)	317	(85.0)	56	(15.0)	
Positive	19	(4.8)	16	(84.2)	3	(15.8)	
MSI status	386/6						0.198^a^
Negative (MSS)	355	(92.0)	298	(83.9)	57	(16.1)	
Positive (MSI)	31	(8.0)	29	(93.5)	2	(6.5)	
OS (months), total	380/12						<0.001^c^
Total/events/censored	380/299/76		321/245/76		59/54/5		
Median survival	14.0		16.7		5.8		
95% confidence interval	11.9–16.1		13.3–20.1		1.3–10.3		
OS (months), pT3/pT4							0.005^c^
Total/events/censored	298/256/42		243/206/37		55/50/5		
Median survival	12.6		13.4		7.3		
95% CI	11.2–13.9		11.1–15.6		1.8–12.8		
OS (months), pT1b/pT2							<0.001^c^
Total/events/censored	82/43/39		78/39/39		4/4/0		
Median survival	50.5		51.7		0.4		
95% CI	36.9–64.0		40.3–63.2		0.0–4.3		
CSS (months), total	353/39						<0.001^c^
Total/events/censored	353/241/112		296/191/105		57/50/7		
Median survival	16.4		18.8		7.3		
95% confidence interval	13.3–19.4		13.7–23.8		1.9–12.6		
CSS (months), pT3/pT4							0.002^c^
Total/events/censored	280/215/65		226/168/58		54/47/7		
Median survival	13.2		14.7		9.0		
95% CI	11.2–15.3		11.4–17.9		3.4–14.6		
CSS (months), pT1b/pT2							<0.001^c^
Total/events/censored	73/26/47		70/23/47		3/3/0		
Median survival	56.0		64.9		4.1		
95% CI	14.1–97.8		18.9–110.9		0.0–10.0		

^a^Fisher’s exact test

^b^Kendall’s tau test

^c^Log-rank test

*UICC* Union for International Cancer Control, *OS* overall survival, *CSS* cancer-specific survival, *CI* confidence interval

^*^Statistically non-significant after multiple testing correction

### Survival analysis

**Fig. 2:**
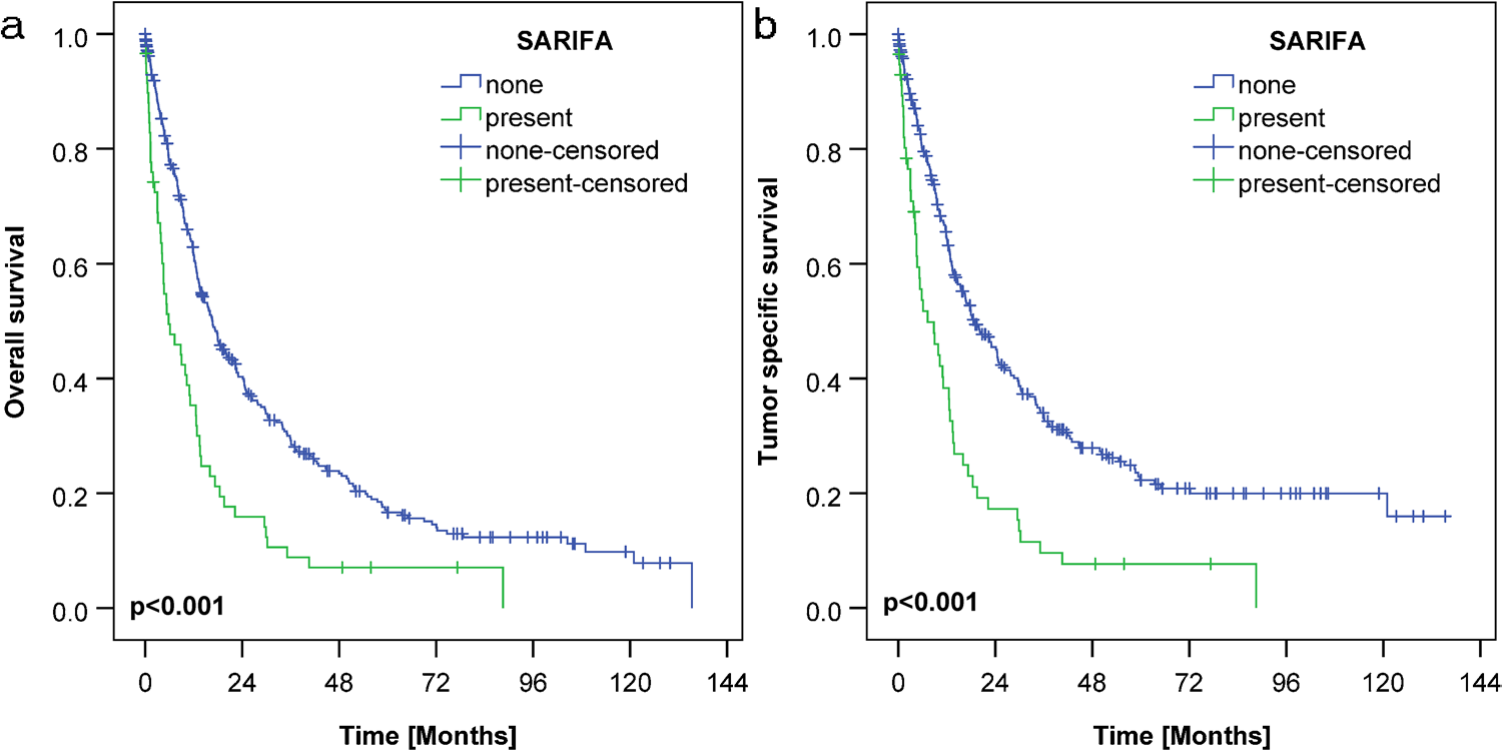
Kaplan–Meier curves representing overall survival (**a**) and cancer-specific survival (**b**) according to SARIFA status in gastric cancer. Note the significantly worse survival in patients with SARIFA-positive tumours (*p* < 0.001, log-rank test)

The median overall survival of the entire cohort was 14.7 months, and the median cancer-specific survival was 16.7 months. Patients with SARIFA-positive GC had shorter median OS and CSS (5.8 months and 7.3 months, respectively) when compared with SARIFA-negative cancers (OS, 16.7 months; CSS, 18.8 months; *p* < 0.001; Fig. 2). Among locally advanced GCs (pT3/pT4; *n* = 298), the median OS was 12.6 months, and the median CSS was 13.2 months. Similarly, as in the whole cohort, SARIFA-positive cases showed shorter median OS (7.3 months, SARIFA-positive vs. 13.4 months, SARIFA-negative; *p* = 0.005) and CSS (9.0 months vs. 14.7 months; *p* = 0.002). There was a very striking difference in OS and CSS between SARIFA-positive and negative early invasive GCs (pT1b/pT2); however, these data should be interpreted with caution due to the low number of cases (see Table 1).

When performing univariate analysis (data not shown), the following variables were identified as potential candidates for the multivariate model: Lauren phenotype, pT, pN, pM, lymph node ratio, pR, LVI, tumour budding, SARIFA, MET and MSI status. To determine whether the SARIFA status affects patients’ CSS, a multivariate Cox regression analysis was performed (see Table 2). There was a significant survival difference between patients with SARIFA-negative and SARIFA-positive GCs (HR = 1.847, 95% CI: 1.300–2.624, *p* = 0.001).

**Table 2:** Multivariate analysis: independent predictors for cancer-specific survival using a Cox proportional hazards model

Variable	HR	95% CI	*p*-value
Laurén phenotype			NS
pT			NS
pN			0.005
pN1 vs. pN0	2.329	1.351–4.015	0.002
pN2 vs. pN0	1.729	0.902–3.314	0.099
pN3 vs. pN0	2.406	1.164–4.976	0.018
M (M1 vs. M0)	1.766	1.221–2.554	0.003
Lymph node ratio (high vs. low)	1.899	1.035–3.485	0.039
R status (pR1/2 vs. pR0)	2.602	1.741–3.887	<0.001
Lymphovascular invasion			NS
Venous invasion			NS
Tumour budding			NS
SARIFA (positive vs. negative)	1.847	1.300–2.624	0.001
MET status (positive vs. negative)	1.874	1.127–3.117	0.015
MSI status (MSI vs. MSS)	0.466	0.227–0.957	0.038

Variables with *p* < 0.100 by univariate analysis were included in the model

*HR* hazard ratio, *CI* confidence interval, *NS* not statistically significant

## Discussion

Recently, a specific histological pattern of direct tumour-adipocyte interaction, named SARIFA (Stroma AReactive Invasion Front areas), was proposed as a promising histology-based prognostic factor in CRC and GC. Similarly, the tumour-adipose feature (TAF) has been identified as a histological feature the machine learning system had used to predict disease-specific survival in CRC [7]. The current study shows that SARIFA is an independent poor prognostic factor in GC.

Grosser et al. demonstrated upregulation of FABP4 and CD36 expression in SARIFA-positive cases [6]. Both FABP4 and CD36 have been suggested as emerging therapeutic targets for cancer [19, 20]. Thus, SARIFA status might serve as a morphology-based biomarker to select patients who may benefit from such targeted treatments without using any additional immunohistochemical or molecular assays.

Several morphology-based biomarkers have been proposed in GC, including poorly differentiated clusters (PDC), tumour budding, stromal maturity or tumour-stroma ratio. According to the definition of SARIFA, a partial overlap exists with PDC, defined as aggregates of at least five cancer cells lacking a gland-like structure [21, 22]. In CRC, Ueno et al. demonstrated that a grading system based on the number of PDC stratified CRC patients by their outcome more effectively than a conventional grade based on loss of gland formation [22]. However, Martin et al. did not detect a statistically significant correlation between SARIFA and PDC in their subcohort of 49 CRC [5], probably due to the small sample size. We previously showed that tumour budding, defined as the presence of single cells or cell clusters of up to four cells in the invasion margin, is associated with various clinicopathological features in GC [17]. Despite that, tumour budding did not retain significance in multivariate analysis. In the current study, SARIFA-positive cases showed more frequent tumour budding; however, the correlation was very weak, supporting the concept that these two histological phenomena have distinct pathogenetic backgrounds. Interestingly, the study by Kemi et al. revealed that stromal immaturity (myxoid changes and keloid-like collagen) and low tumour-stroma ratio (i.e. high proportion of stroma) are both independent adverse prognostic factors in GC [3, 4]. The relationship between poor prognosis and both (1) SARIFA and (2) high stromal volume warrants further research to determine if a stroma-rich GC can demonstrate SARIFA areas or if they are mutually exclusive morphological features.

SARIFA were observed mostly in advanced GC stages, which is consistent with earlier findings [6, 23, 24]. This could explain shorter OS and CSS when compared to SARIFA-negative cases. Nevertheless, subgroup analysis of pT3/pT4 GCs revealed similar results. Moreover, the difference in survival data was also observed in early invasive tumours, although there were only 4 SARIFA-positive pT1b/pT2 cases in the cohort; thus, these data should be interpreted with caution.

The current results showing a relatively high number of SARIFA-positive cases in *MET*-amplified GCs may warrant further studies of larger cohorts. *MET* activation has been linked to epithelial-mesenchymal transition and various cellular processes of invasive growth like motility, survival, proliferation, morphogenesis and angiogenesis [25]. *MET* amplification in GC has been associated with more aggressive tumour phenotypes, advanced stages and shorter survival, as previously demonstrated by this and other collectives [12, 26].

Recently, Grosser et al. showed that SARIFA status could have a prognostic role in patients who have received perioperative chemotherapy. By assessing SARIFA status in cohorts of MAGIC and ST03 trials, they demonstrated that SARIFA status can identify patients with poor prognosis when assessed in the post-chemotherapy resection specimens from the ST03 trial patients, but not from MAGIC trial patients [24]. As multimodal treatment has become a standard of care in GC, additional validating studies are needed to verify the role of SARIFA as a prognostic biomarker in neoadjuvant-treated GC.

This study has some limitations. It is a retrospective study, and the assessment is carried out on previously collected single tissue sections. One tissue slide per case might not be representative and could lead to sampling bias; however, the same approach was used in past studies. Another limitation is the unclear definition of invasive front, specifically its width. Several approaches exist in the literature, including the most distant cell layers or measured thickness, e.g. 250 μm, 500 μm or 1000 μm. Here, the authors evaluated the outermost cell layers at the invading edge of the tumour. This could explain the relatively low number of SARIFA-positive cases in the current study when compared to the assessment carried out by Grosser et al. [23]; however, SARIFA status appears to be such a robust prognostic factor that different assessment methods might give similar prognostic information.

In conclusion, the results of this study show that SARIFA is strongly indicative of adverse prognosis in primary resected, i.e. chemotherapy-naive GC, and may be suitable to tailor adjuvant patient management. Assessment of SARIFA is a simple and cost-effective method to provide relevant prognostic information during routine examination of resection specimens without additional immunohistochemistry, molecular assays or computational approaches. It represents a high-risk GC phenotype and seems to surpass tumour budding as a prognostic factor in GC. Because the evaluation of SARIFA is restricted to resection specimens, further studies of SARIFA-positive GCs are warranted to understand the biology of this “high-risk phenotype.” Additionally, the significance of SARIFA should be studied in other solid cancers.

## References

[1] Sung H, Ferlay J, Siegel RL et al (2021) Global Cancer Statistics 2020: GLOBOCAN estimates of incidence and mortality worldwide for 36 cancers in 185 countries. CA Cancer J Clin 71:209–249. 10.3322/caac.21660 33538338 10.3322/caac.21660

[2] Szalai L, Jakab Á, Kocsmár I et al (2022) Prognostic ability of tumor budding outperforms poorly differentiated clusters in gastric cancer. Cancers (Basel) 14. 10.3390/cancers14194731 10.3390/cancers14194731 PMC9563769 36230653

[3] Kemi NA, Eskuri M, Pohjanen V-M et al (2019) Histological assessment of stromal maturity as a prognostic factor in surgically treated gastric adenocarcinoma. Histopathology 75:882–889. 10.1111/his.13934 31173384 10.1111/his.13934

[4] Kemi N, Eskuri M, Herva A et al (2018) Tumour-stroma ratio and prognosis in gastric adenocarcinoma. Br J Cancer 119:435–439. 10.1038/s41416-018-0202-y 30057407 10.1038/s41416-018-0202-y PMC6133938

[5] Martin B, Grosser B, Kempkens L et al (2021) Stroma AReactive Invasion Front Areas (SARIFA)-a new easily to determine biomarker in colon cancer-results of a retrospective study. Cancers (Basel) 13:4880. 10.3390/cancers13194880 34638364 10.3390/cancers13194880 PMC8508517

[6] Grosser B, Glückstein M-I, Dhillon C et al (2022) Stroma AReactive Invasion Front Areas (SARIFA) - a new prognostic biomarker in gastric cancer related to tumor-promoting adipocytes. J Pathol 256:71–82. 10.1002/path.5810 34580877 10.1002/path.5810

[7] Wulczyn E, Steiner DF, Moran M et al (2021) Interpretable survival prediction for colorectal cancer using deep learning. npj Digital Medicine 4:71. 10.1038/s41746-021-00427-2 33875798 10.1038/s41746-021-00427-2 PMC8055695

[8] Mukherjee A, Bilecz AJ, Lengyel E (2022) The adipocyte microenvironment and cancer. Cancer Metastasis Rev 41:575–587. 10.1007/s10555-022-10059-x 35941408 10.1007/s10555-022-10059-x

[9] Ma X, Xiao L, Liu L et al (2021) CD36-mediated ferroptosis dampens intratumoral CD8+ T cell effector function and impairs their antitumor ability. Cell Metab 33:1001–1012.e5. 10.1016/j.cmet.2021.02.015 33691090 10.1016/j.cmet.2021.02.015 PMC8102368

[10] Brierley JD, Gospodarowicz MK, Wittekind C (2017) TNM classification of malignant tumours. John Wiley & Sons

[11] Warneke VS, Behrens H-M, Böger C et al (2013) Her2/neu testing in gastric cancer: evaluating the risk of sampling errors. Ann Oncol 24:725–733. 10.1093/annonc/mds528 23139264 10.1093/annonc/mds528 PMC3574551

[12] Metzger M-L, Behrens H-M, Böger C et al (2016) MET in gastric cancer--discarding a 10% cutoff rule. Histopathology 68:241–253. 10.1111/his.12745 26033401 10.1111/his.12745 PMC4744765

[13] Mathiak M, Warneke VS, Behrens H-M et al (2017) Clinicopathologic characteristics of microsatellite instable gastric carcinomas revisited: urgent need for standardization. Appl Immunohistochem Mol Morphol 25:12–24. 10.1097/PAI.0000000000000264 26371427 10.1097/PAI.0000000000000264 PMC5147042

[14] Böger C, Krüger S, Behrens HM et al (2017) Epstein-Barr virus-associated gastric cancer reveals intratumoral heterogeneity of PIK3CA mutations. Ann Oncol 28:1005–1014. 10.1093/annonc/mdx047 28453696 10.1093/annonc/mdx047 PMC5406766

[15] Zlobec I, Bächli M, Galuppini F et al (2021) Refining the ITBCC tumor budding scoring system with a “zero-budding” category in colorectal cancer. Virchows Arch 479:1085–1090 33843013 10.1007/s00428-021-03090-w PMC8724067

[16] Lugli A, Kirsch R, Ajioka Y et al (2017) Recommendations for reporting tumor budding in colorectal cancer based on the International Tumor Budding Consensus Conference (ITBCC) 2016. Mod Pathol 30:1299–1311 28548122 10.1038/modpathol.2017.46

[17] Ulase D, Heckl S, Behrens H-M et al (2020) Prognostic significance of tumour budding assessed in gastric carcinoma according to the criteria of the International Tumour Budding Consensus Conference. Histopathology 76:433–446. 10.1111/his.13997 31538348 10.1111/his.13997

[18] Benjamini Y, Hochberg Y (1995) Controlling the false discovery rate: a practical and powerful approach to multiple testing. J R Stat Soc Ser B Methodol 57:289–300

[19] Sun N, Zhao X (2022) Therapeutic implications of FABP4 in cancer: an emerging target to tackle cancer. Front Pharmacol 13:948610 35899119 10.3389/fphar.2022.948610 PMC9310032

[20] Ruan C, Meng Y, Song H (2022) CD36: an emerging therapeutic target for cancer and its molecular mechanisms. J Cancer Res Clin Oncol 148:1551–1558. 10.1007/s00432-022-03957-8 35224665 10.1007/s00432-022-03957-8 PMC11801024

[21] Ueno H, Mochizuki H, Hashiguchi Y et al (2008) Histological grading of colorectal cancer: a simple and objective method. Ann Surg 247:811–818 18438118 10.1097/SLA.0b013e318167580f

[22] Ueno H, Kajiwara Y, Shimazaki H et al (2012) New criteria for histologic grading of colorectal cancer. Am J Surg Pathol 36:193–201 22251938 10.1097/PAS.0b013e318235edee

[23] Grosser B, Heyer CM, Austgen J et al (2023) Stroma AReactive Invasion Front Areas (SARIFA) proves prognostic relevance in gastric carcinoma and is based on a tumor–adipocyte interaction indicating an altered immune response. Gastric Cancer 27(1):72–85. 10.1007/s10120-023-01436-8 37874427 10.1007/s10120-023-01436-8 PMC10761465

[24] Grosser B, Emmerson J, Reitsam NG et al (2023) Stroma AReactive Invasion Front Areas (SARIFA) improves prognostic risk stratification of perioperative chemotherapy treated oesophagogastric cancer patients from the MAGIC and the ST03 trial. Br J Cancer 130(3):457–466. 10.1038/s41416-023-02515-4 38123705 10.1038/s41416-023-02515-4 PMC10844337

[25] Hack SP, Bruey J-M, Koeppen H (2014) HGF/MET-directed therapeutics in gastroesophageal cancer: a review of clinical and biomarker development. Oncotarget 5(10):2866 24930887 10.18632/oncotarget.2003 PMC4102777

[26] Lee J, Won SJ, Jung JH et al (2011) Impact of MET amplification on gastric cancer: Possible roles as a novel prognostic marker and a potential therapeutic target. Oncol Rep 25:1517–1524. 10.3892/or.2011.1219 21424128 10.3892/or.2011.1219

